# Editorial: Exercise in the prevention, management of and rehabilitation from cardiovascular diseases

**DOI:** 10.3389/fphys.2023.1255634

**Published:** 2023-07-18

**Authors:** Ferdinando Iellamo

**Affiliations:** Department of Clinical Science and Translational Medicine, University of Rome Tor Vergata, Roma, Italy

**Keywords:** exercise training, cardiovascular disease, non-pharmacological therapy, cardiac rehabilitation, cardiovascular prevention

Management of and Rehabilitation from cardiovascular diseases (CVD) in the recent decades is one of the greatest tasks for health professionals. The Research Topic “*Exercise in the prevention, management of and rehabilitation from cardiovascular diseases*” presents information on the cardiovascular effects of various forms of exercise through original and review articles. All these studies provide different insights into the therapeutic effects of exercise in individuals with CVD.

Although regular physical activity has been proven to reduce CVD associated morbidity and mortality, optimal exercise modality, in terms of type of exercise, duration, frequency, and intensity of it remains unclear. New training protocols were developed and tested in recent years. Alternatives to standard rehabilitation have been proposed. In this Research Topic, the original article developed by Marzuca-Nassr et al., showed that a 12-week hybrid exercise-based cardiac rehabilitation program (i.e., a first face-to-face phase plus a second remote monitoring phase) was capable of improving both functional capacity (6 min walking test) and muscle strength (handgrip) in adults and older patients with coronary disease. New delivery strategies, like hybrid cardiac rehabilitation and tele-rehabilitation as well, could help to improve access and increase adherence to rehabilitation programs in patients with coronary artery disease, especially in the elderly.

Physical exercise is a non-pharmacological strategy not only in patients with cardiac diseases but also in the management of arterial hypertension. To understand better the mechanisms of the blood pressure lowering effect of exercise, an expedient model has been that of studying blood pressure behaviour for a time after a single bout of different exercise modalities, the so-called post exercise hypothension (PEH). By using this approach, Ramis et al. investigated the effects of a single aerobic and resistance exercise sessions on ambuatory blood pressure monitoring (ABPM) in middle-aged subjects with hypertension, and found that PEH did occur in both exercise groups, although it was more pronounced after aerobic exercise. ABPM did not differ between both types of exercising and a control groups sessions for 24 h. This study gives support to the hypothesis that the accumulation of exercise sessions could amplify the beneficial effects of pharmacological treatment (Brito et al., 2018). Overall, evidence from this (Ramis et al.) and other studies would indicate that exercise can lower blood pressure, an effect more evident with the aerobic intervention.

However, the exact mechanisms of exercise benefits are not yet fully known. It would be of great interest to know how physical activity can affect CVD, as preventive and treatment tool.

It is assumed that benefits of exercise include effects on endothelial function, in addition to the effects on autonomic nervous system (Floras et al., 1989) and metabolic pathways (Hussain et al., 1996; Halliwill, 2001). The systematic review and meta-analysis conducted by Tao et al. summarized the currently available information on the effects of different durations and intensities of aerobic exercise on vascular endothelial function in different populations, starting from the assumpion that the duration and the intensity of exercise are the key factors that affect the endothelial function, as assessed by flow-mediated dilation technique (FMD). The review included 20 RCT studies and the results indicated that moderate- and vigorous-intensity, but not low-intensity, aerobic exercise, improved FMD, an effect associated with the duration of the training program (i.e., the longer the better). In addition, the improvement was greater in patients with a worse basal FMD and of older age.

Further studies are clearly warranted to understand the optimal exercise protocols and the underlying mechanisms.

Within this framework, alternative training modalities, in addition to the classic aerobic and resistance types of exercises, have been also tested. Among these, Han and Ju investigated the effects of an ancient martial art, the taekwondo, on measures of metabolic syndrome (MS), that included Body Mass Index (BMI), Systolic (BP) and Diastolic (DBP) Blood Pressure, Fasting Blood Glucose (FBG), Trygliceride (TG) and High Density Lipoproteins-Cholesterol (HDL-C) (Grundy, 2016). Han and Ju carried out a thorough meta-analysis of 45 studies, most of which coming from South Korea, comprising 1,079 subjects overall, that used taekwondo as the exercise paradigm. The authors (Han and Ju) reported that all MS indicators significantly improved with the taekwondo intervention, similarly to the more classic aerobic (Ostman et al., 2017), endurance (Pattyn et al., 2013) and resistance training (Wewege et al., 2018).

The authors ascribed these effects to the taekwondo training characteristics, which involve complicated movements that require the synchronization of numerous body parts, such as twisting blocks, flying kicks, and fast motions, not restricted to just one portion of the body, resembling most of all other exercise training modalities. The beneficial effects of taekwondo on the MS indicators were more accentuated in older woman population, exercising three times a week for at least 12 weeks at moderate to high intensity, similarly to the recommendations of the American Diabetes Associaton (Arnett et al., 2019; American Diabetes Association, 2020).

However, given the specificity of taekwondo training and its mainly regional diffusion, these benefits, at present, cannot be generalized to the general population around the world.

Overall, the research discussed in this Research Topic underscores the importance of exercise as a major component in preventing and managing CVD. It highlights the effectiveness of various exercise training strategies in improving many cardiovascular parameters and risk factors with the potential of preventing the development of CVD.

## References

[B1] American Diabetes Association (2020). 5. Facilitating behavior change and well-being to improve health outcomes: *Standards of medical Care in diabetes-2020* . Diabetes Care 43, S48–S65. 10.2337/dc20-S005 31862748

[B2] Arnett D. K. Blumenthal R. S. Albert M. A. Buroker A. B. Goldberger Z. D. Hahn E. J. (2019). 2019 ACC/AHA guideline on the primary prevention of cardiovascular disease: Executive summary: A report of the American college of cardiology/American heart association Task Force on Clinical practice guidelines. J. Am. Coll. Cardiol. 74, 1376–1414. 10.1016/j.jacc.2019.03.009 30894319 PMC8344373

[B3] Brito L. C. Fecchio R. Y. Pecanha T. Andrade-Lima A. Halliwill J. R. Forjaz C. L. M. (2018). Postexercise hypotension as A clinical tool: A "single brick" in the wall. J. Am. Soc. Hypertens. 12, E59–E64. 10.1016/j.jash.2018.10.006 30425018

[B4] Floras J. S. Sinkey C. A. Aylward P. E. Seals D. R. Thorén P. N. Mark A. L. (1989). Postexercise hypotension and sympathoinhibition in borderline hypertensive men. Hypertension 14, 28–35. 10.1161/01.hyp.14.1.28 2737735

[B5] Grundy S. M. (2016). Metabolic syndrome update. Trends cardiovasc. Med. 26, 364–373. 10.1016/j.tcm.2015.10.004 26654259

[B6] Halliwill J. R. (2001). Mechanisms and clinical implications of postexercise hypotension in humans. Exerc Sport Sci. Rev. 29, 65–70. 10.1097/00003677-200104000-00005 11337825

[B7] Hussain S. T. Smith R. E. Medbak S. Wood R. F. Whipp B. J. (1996). Haemodynamic and metabolic responses of the lower limb after high intensity exercise in humans. Exp. Physiol. 81, 173–187. 10.1113/expphysiol.1996.sp003923 8845133

[B8] Ostman C. Smart N. Morcos D. Duller A. Ridley W. Jewiss D. (2017). The effect of exercise training on clinical outcomes in patients with the metabolic syndrome: A systematic review and meta-analysis. Cardiovasc Diabetol. 16, 110. 10.1186/s12933-017-0590-y 28854979 PMC5577843

[B9] Pattyn N. Cornelissen V. Eshghi S. Vanhees L. (2013). The effect of exercise on the cardiovascular risk factors constituting the metabolic syndrome A meta-analysis of controlled trials. Sports Med. 43, 121–133. 10.1007/s40279-012-0003-z 23329606 PMC3693431

[B10] Wewege M. Thom J. Rye K. Parmenter B. (2018). Aerobic, resistance or combined training: A systematic review and meta-analysis of exercise to reduce cardiovascular risk in adults with metabolic syndrome. Atherosclerosis 274, 162–171. 10.1016/j.atherosclerosis.2018.05.002 29783064

